# Supplementary material for: Microbe-host interplay in atopic dermatitis and psoriasis
Source: Nat Commun. 2019 Oct 16;10:4703. doi: 10.1038/s41467-019-12253-y (PMC6795799; doi:10.1038/s41467-019-12253-y)
Supplement: Supplementary file 1 — Supplementary Information [file 41467_2019_12253_MOESM1_ESM.pdf]

Microbe-host interplay in atopic dermatitis and psoriasis

Fyhrquist *et al*

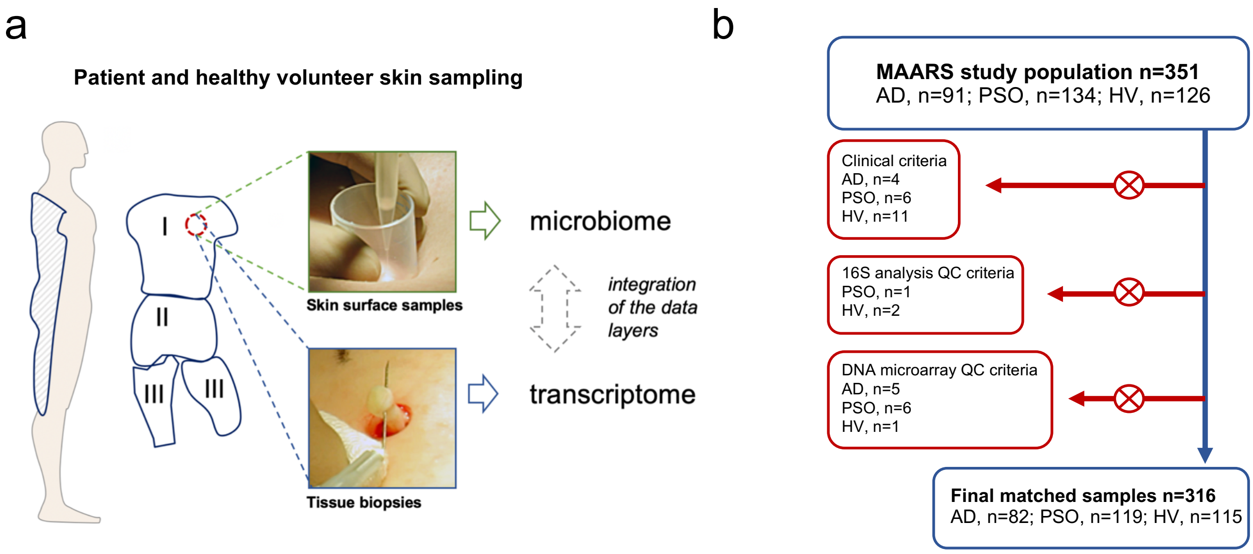

c

	Atopic Dermatitis	Psoriasis	Healthy Volunteers
Numbers	82	119	115
Age			
Mean ± SEM	44.49 ± 1.59	48.8 ± 1.23	34.9 ± 1.17
Range	20 - 83	20 - 77	19 - 77
Sex (M/F)	46/36	93/26	44/71
SCORAD	50.6 ± 1.93	-	-
PASI	-	14.09 ± 0.79	-

Supplementary Figure 1. Patient sample collection. Patient **a** sampling and **b** enrollment flow chart. **c** Clinical data. The source data files used to generate the present figure are available from the NCBI Sequence Read Archive under accession PRJNA554499, and from EBI ArrayExpress under accession E-MTAB-8149.

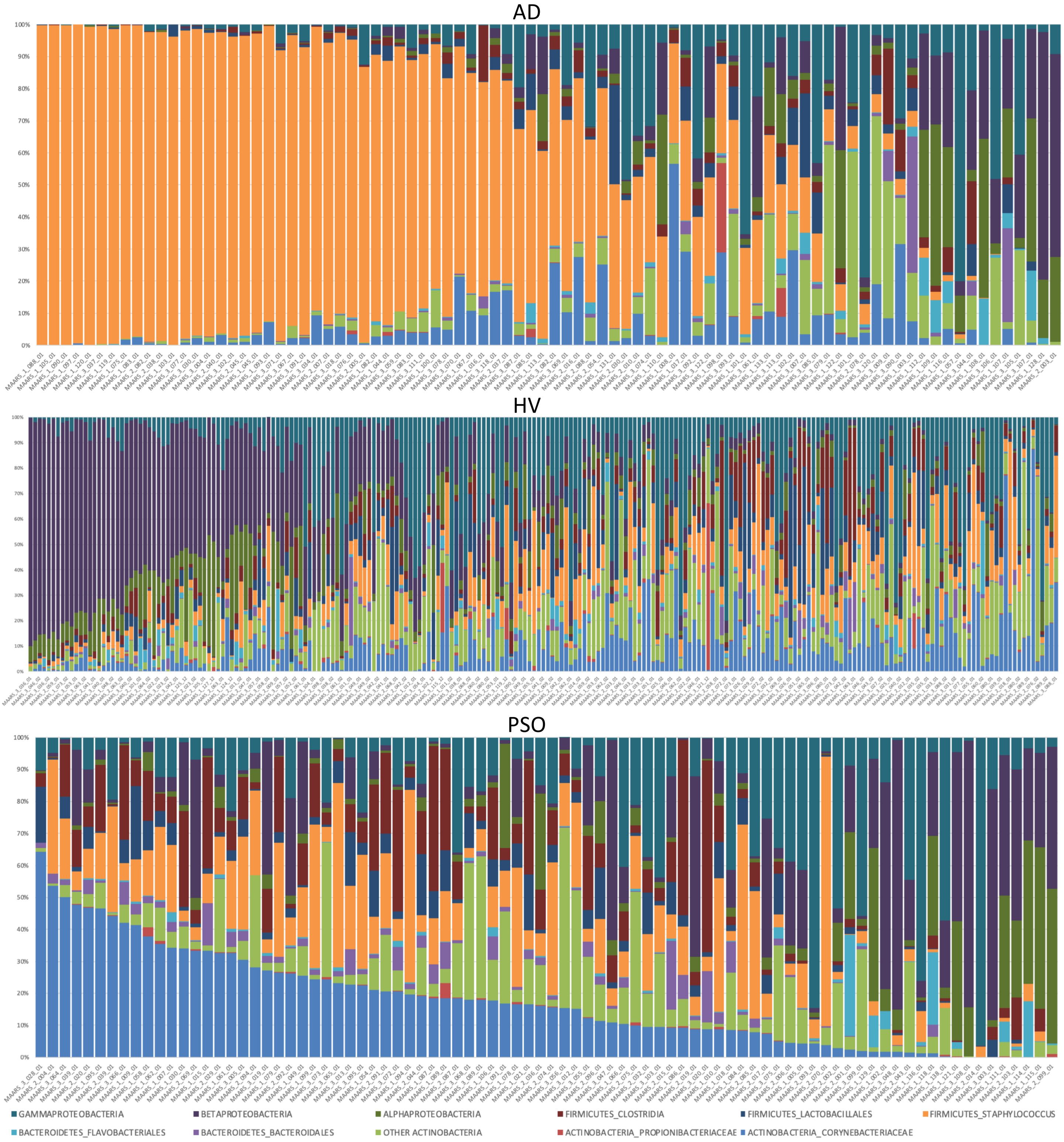

Supplementary Figure 2. Individual compositions of the skin microbiota in AD, HV and PSO. The most abundant bacterial groups depicted for HV, AD and PSO based on 16S rRNA gene sequences. The source data files used to generate the present figure are available from the NCBI Sequence Read Archive under accession PRJNA554499.

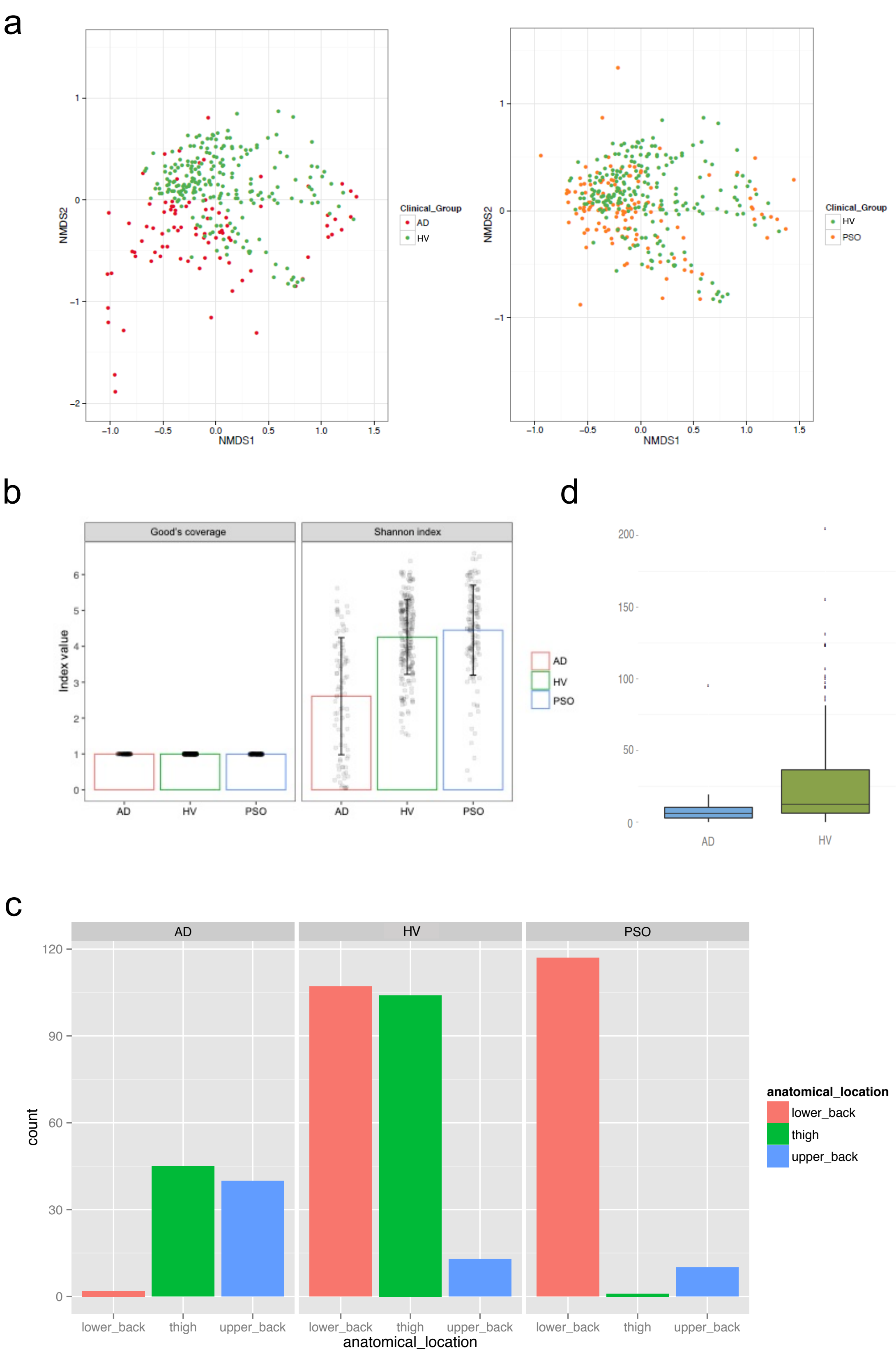

Supplementary Figure 3. Characterization of the skin microbiota in AD and PSO. a NMDS analysis of the 95 most abundant OTUs in the three patient groups (HV, *n*=115; AD, *n*=82; PSO, *n*=119). **b** Average Good's coverage and Shannon diversity index in the three sample sets. **c** Sampling from different skin sites in the three different clinical groups, revealing the most significant potential confounding factor. (HV lower back, *n*=107, thigh, *n*=104, upper back, *n* =13; AD, lower back, *n*=2, thigh, *n*=44 upper back, *n* =40; PSO, lower back, *n*=107, thigh, *n*=1, upper back , *n* =10) **d** Anaerobe abundance in AD and HV samples without *S. aureus* (HV, *n*=115, AD, *n*=82, Mann-Whitney U test, *p*-value 0.0003662) The center line in the boxplots corresponds to the median, the bounding box is the interquantile range (IQR) and the whiskers are defined as 1.5 times IQR. The source data files used to generate the present figure are available from the NCBI Sequence Read Archive under accession PRJNA554499.

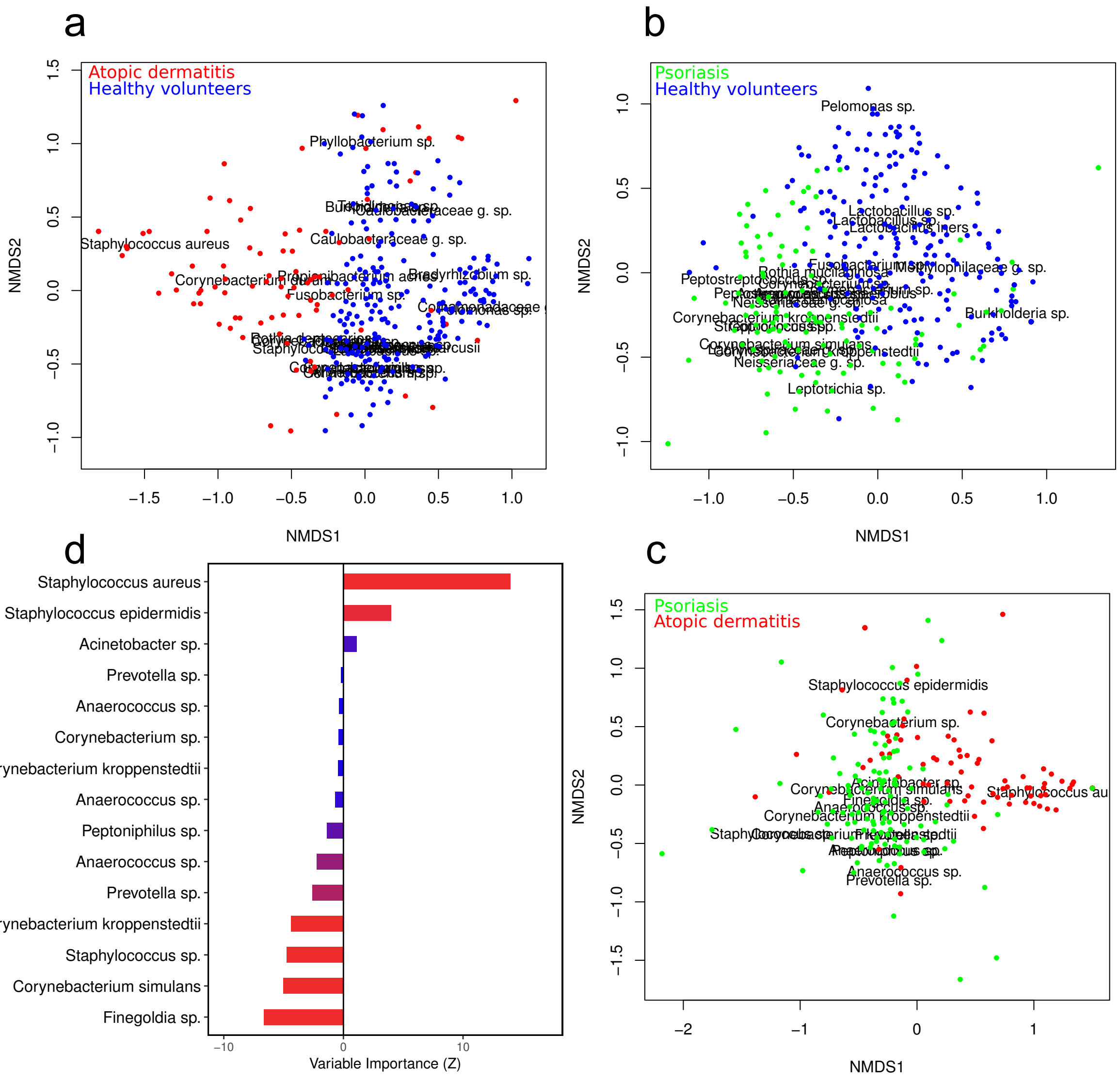

Supplementary Figure 4. Microbiota based classification of AD and PSO. NMDS analysis considering only microbes identified through feature selection. **a** HV (*n*=115) vs AD (*n*=82), **b** HV vs PSO (*n*=119). **c** Variable importance for the best set of discriminatory taxa identified through Random Forest feature selection (Boruta) and classification analysis for AD vs PSO. **d** PSO vs AD. The source data files used to generate the present figure are available from the NCBI Sequence Read Archive under accession PRJNA554499.

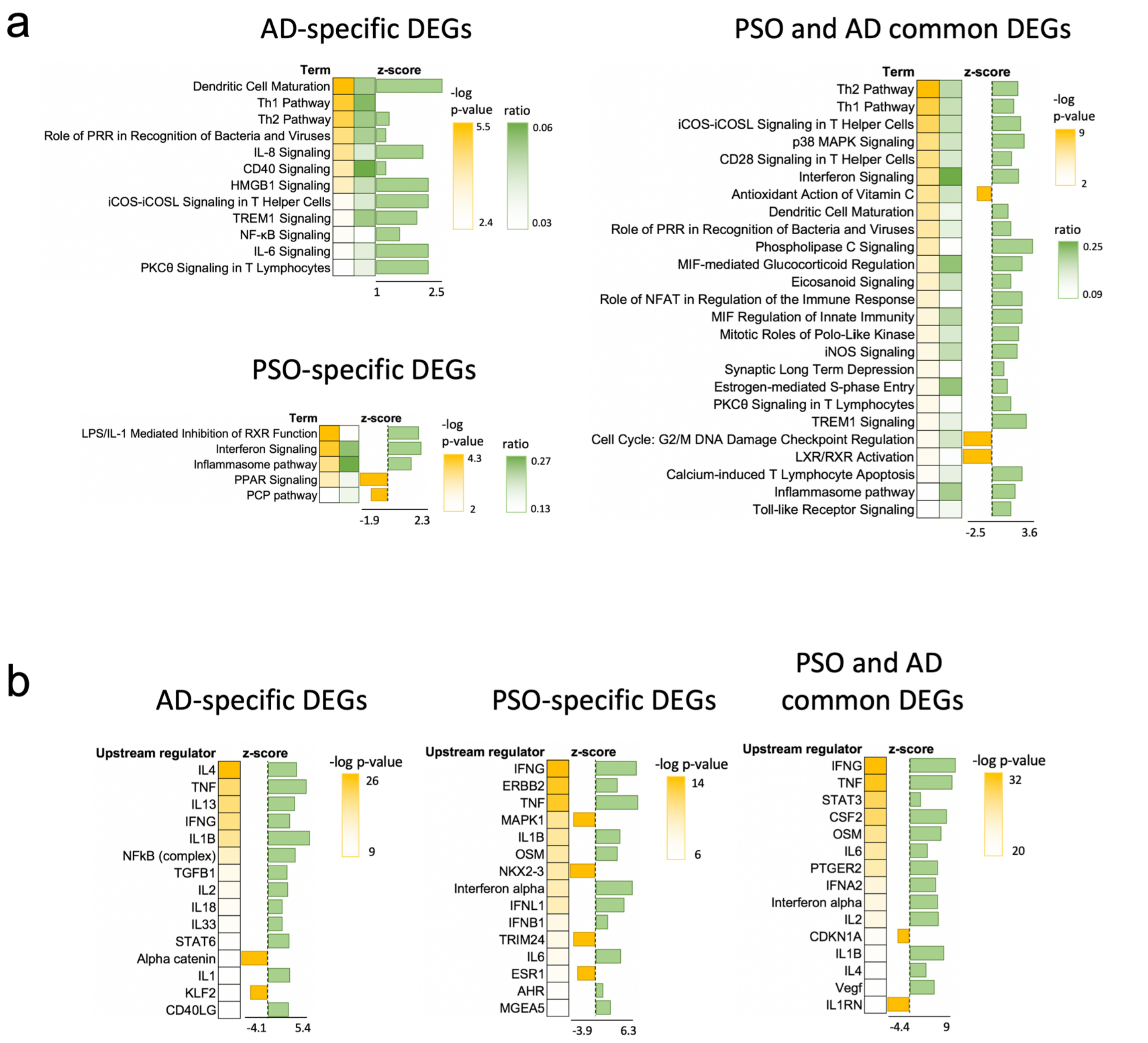

Supplementary Figure 5. The AD and PSO skin transcriptomes. a Most significantly enriched pathways (-log *p*-value >2, z-score >1 or <-1) according to IPA analysis of AD (*n*=82) and PSO (*n*=119) specific and common genes. **b** Top 15 upstream regulators (-log *p*-value >2, z-score >1 or <-1) predicted by IPA. The source data files used to generate the present figure are available from EBI ArrayExpress under accession E-MTAB-8149.

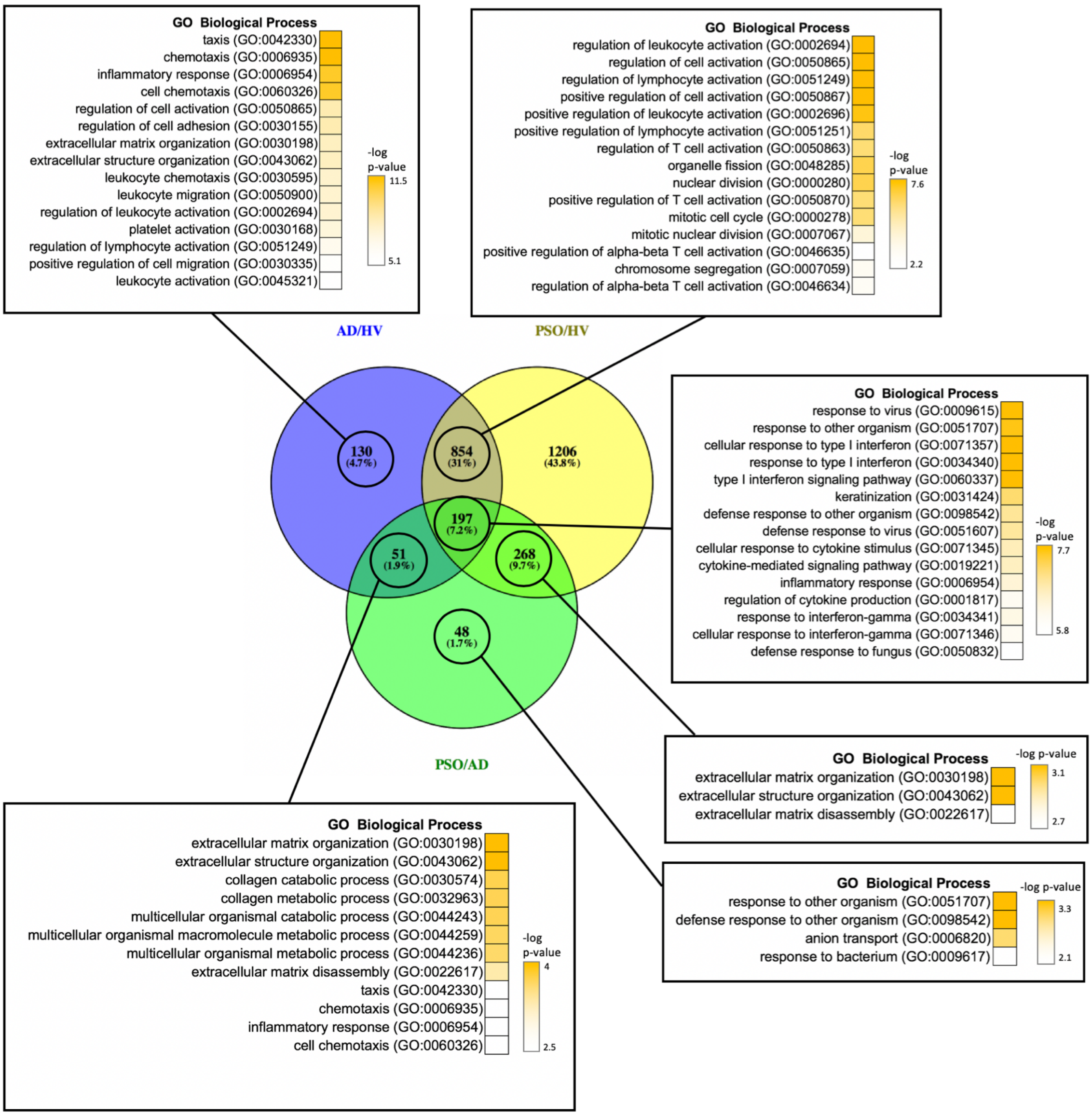

Supplementary Figure 6. Common and distinct functions in AD and PSO skin transcriptomes. GO Term analysis of Venn diagram subsections of AD (*n*=82) and PSO (*n*=119) vs HV (*n*= 115) differentially expressed genes; GO Term Biological Process, -log *p*-value >2. The source data files used to generate the present figure are available from EBI ArrayExpress under accession E-MTAB-8149.

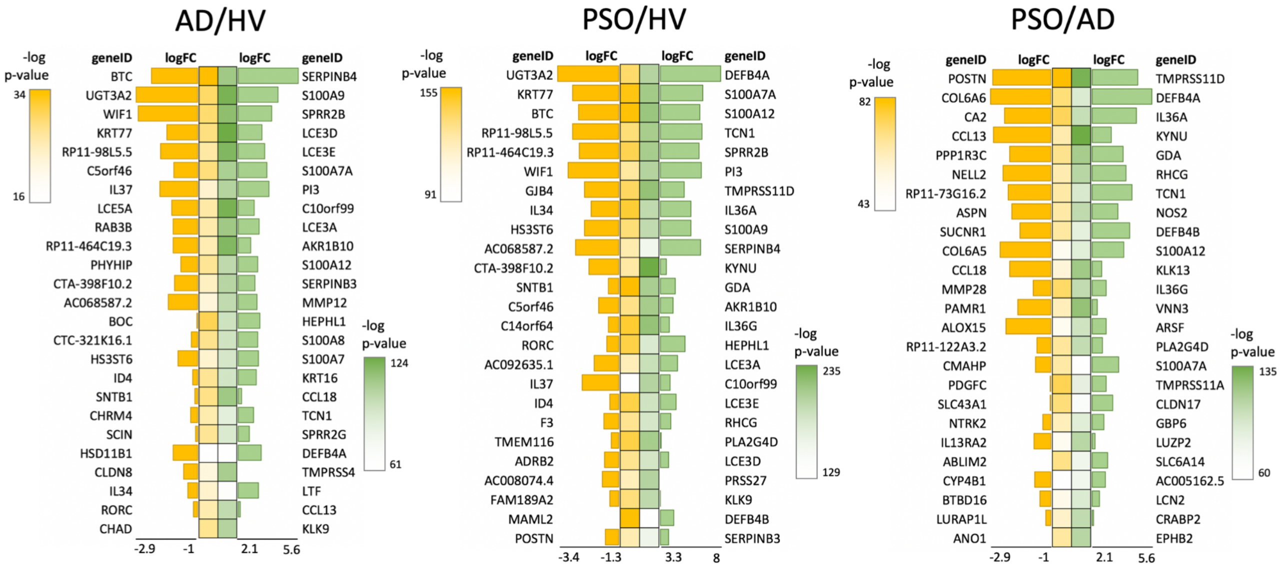

Supplementary Figure 7. Top genes in AD and PSO skin transcriptomes. Top 25 up- and downregulated genes organized according to Rank-value (-log *p*-value * logFC). The source data files used to generate the present figure are available from EBI ArrayExpress under accession E-MTAB-8149.

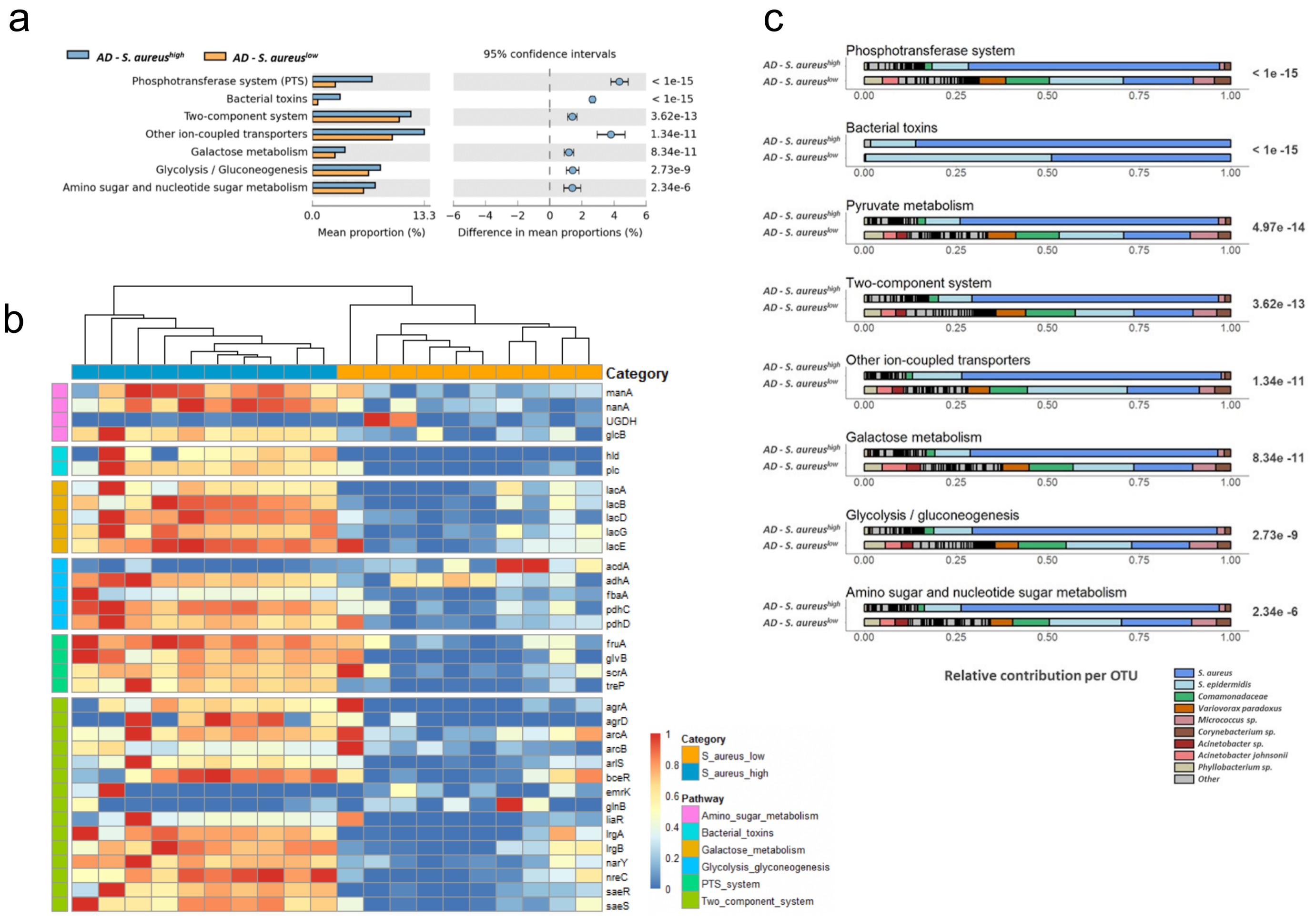

Supplementary Figure 8. Prediction and validation of the *S. aureus* metagenome. a Significantly differentially enriched microbial pathways between *S. aureus* 'high' (*n*=27) and 'low' (*n*=25) groups. Gene content of individual OTUs was inferred using the Greengenes v.13.5 database, and subsequently used to predict enriched microbial pathways in the respective disease groups. **b** WGS metagenomic abundances of top differentially abundant genes between *S. aureus* 'high' (blue) and *S. aureus* 'low' (orange) cohorts, annotated by KEGG pathway categories. **c** Contribution of individual OTUs to relevant microbial pathways. The gene content of individual OTUs was inferred using the Greengenes v.13.5 database, and subsequently used to predict enriched microbial pathways in the respective disease groups. Horizontal bars represent the percentage of genes contributed by the most abundant microbes in the dataset. X-axis: sum of relative contributions per sample. Y-axis: contribution across *S. aureus* 'high' and *S. aureus* 'low' samples, respectively. The source data files used to generate the present figure are available from the NCBI Sequence Read Archive under accession PRJNA554499.

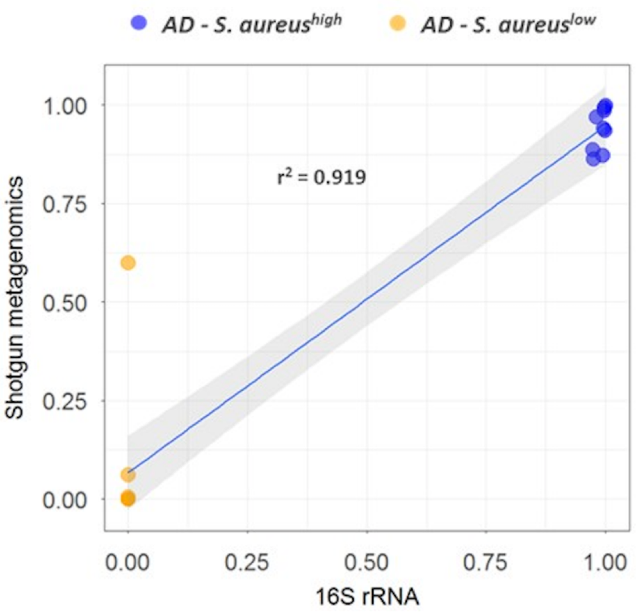

Supplementary Figure 9. Correlation between 16S rRNA gene sequencing and WGS metagenomics data. For validation of 16S OTU classification, preliminary data of WGS metagenomic sequencing of 20 randomly selected samples from 10 *S. aur*eus ‘high’ and 10 *S. aureus* ‘low’ AD lesions were used. The significant correlation (r2=0.919) shows agreement of taxonomic classification between the independent sequencing methods. The source data files used to generate the present figure are available from the NCBI Sequence Read Archive under accession PRJNA554499.

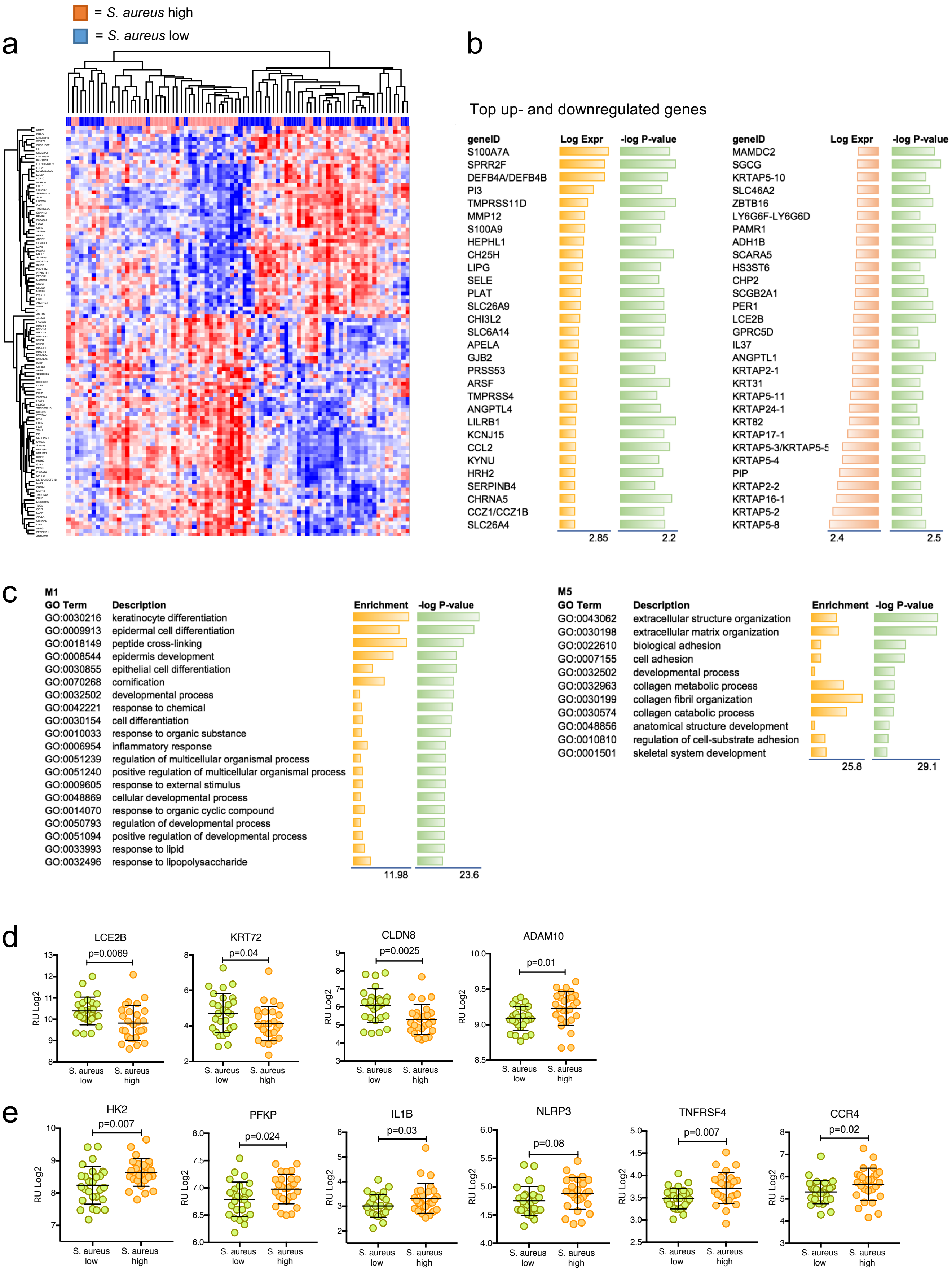

Supplementary Figure 10. Further analysis of the *S. aureus* signature. a Hierarchical clustering of differentially expressed host genes between *S. aureus* 'high' (*n*=27) and 'low' (*n*=25) abundance samples (left panel), and **b** a list of top up- and downregulated DEGs. **c** Gene ontology based analysis of biological functions enriched in M1 and M5 modules in the AD gene co-expression network. Statistical analysis (Mann-Whitney U-test) of **d** barrier associated genes, and **e** HIF1A dependent genes, proinflammatory and Th2 type signature genes in *S. aureus* 'high' and 'low' samples. The center line in the dot plots corresponds to the mean, and the error bars to the standard deviation. The source data files used to generate the present figure are available from the NCBI Sequence Read Archive under accession PRJNA554499, and from EBI ArrayExpress under accession E-MTAB-8149.

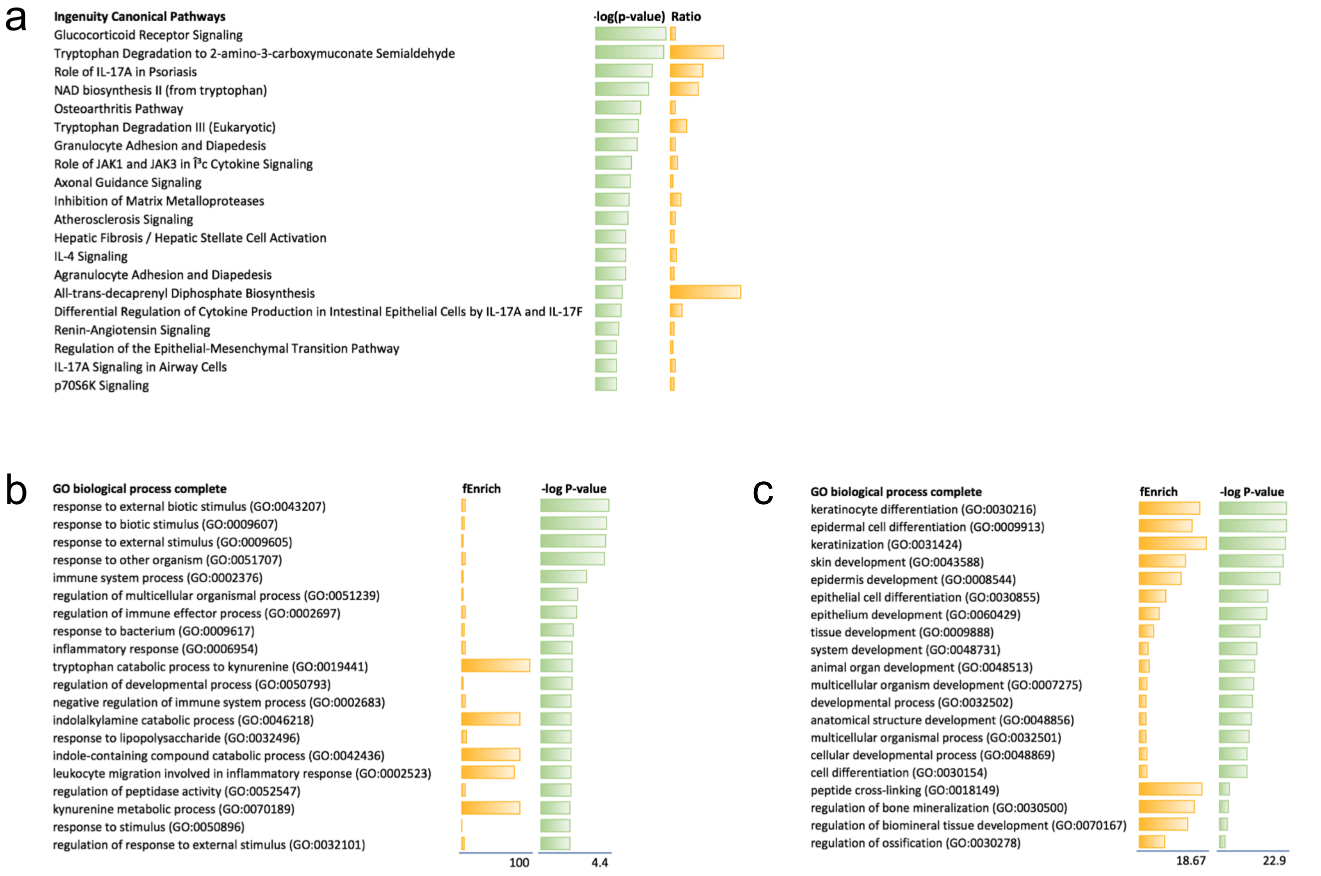

Supplementary Figure 11. Functional enrichment in the *S. aureus* gene signature. a Identification of biological function in the *S. aureus* signature (comparing *S. aureus* 'high' (*n*=27) and 'low' (*n*=25) abundance samples), using Ingenuity Pathway analysis (IPA), and **b** Gene ontology based analysis of functional enrichment among up-regulated and **c** down-regulated *S. aureus* signature genes. The source data files used to generate the present figure are available from the NCBI Sequence Read Archive under accession PRJNA554499, and from EBI ArrayExpress under accession E-MTAB-8149.

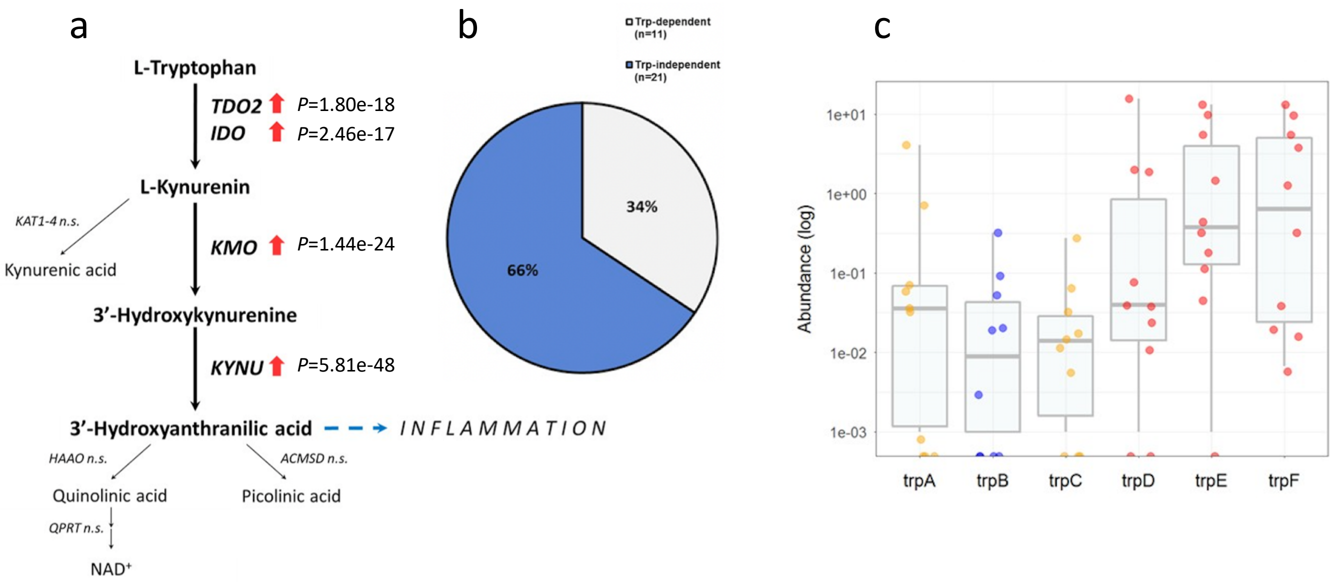

Supplementary Figure 12. Inference of host and microbial functional features. a Regulation of the kynureninase pathway of trp degradation on the mRNA level between the *S. aureus* 'high' (*n*=27) and 'low' (*n*=25) cohorts. Red arrows: significantly regulated genes, n.s.: not significant. Arrow and font thickness correspond to significance. **b** Culture-based trp dependence assay of 32 *S. aureus* strains isolated from moderate-to-severe atopic dermatitis patients. Overall, 66% of colonizing *S. aureus* strains were shown to grow independent of trp in trp-depleted culture medium. **c** Presence of tryptophan biosynthesis-related gene families in WGS sequencing results of *S. aureus* 'high' samples. Y axis: relative abundance of UniRef50-defined trp gene families. Boxplots represent the median (center line), interquartile range (hinges) and 1.5x interquartile range (whiskers). The source data files used to generate the present figure are available from the NCBI Sequence Read Archive under accession PRJNA554499, and from EBI ArrayExpress under accession E-MTAB-8149.

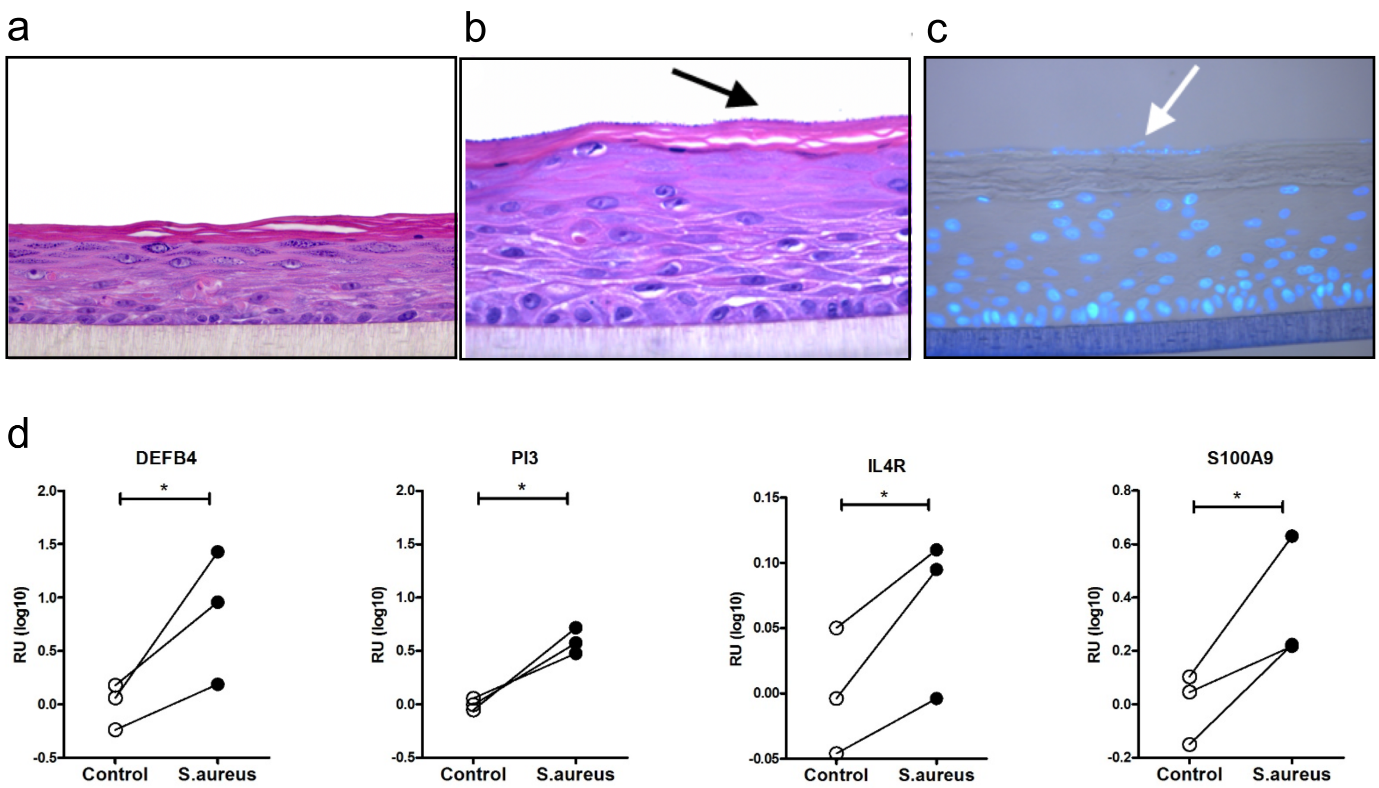

Supplementary Figure 13. Microbial stimulation of human epidermal equivalents (HEEs) a Morphological analysis of HEEs. **b** Hematoxylin Eosin (HE) and **c** DAPI staining of microbial colonization of HEEs cultured for 8 days at the air-liquid interface. *S. aureus* bacteria were exposed to the skin equivalent. Arrows indicate visible bacteria on top of the stratum corneum. **d** Statistical analysis (n=3, paired one-sided Student's t-test, p<0.05) of gene expression after bacterial stimulation; qPCR measurement of selected *S. aureus* signature genes. The source data are provided as a Source Data file.

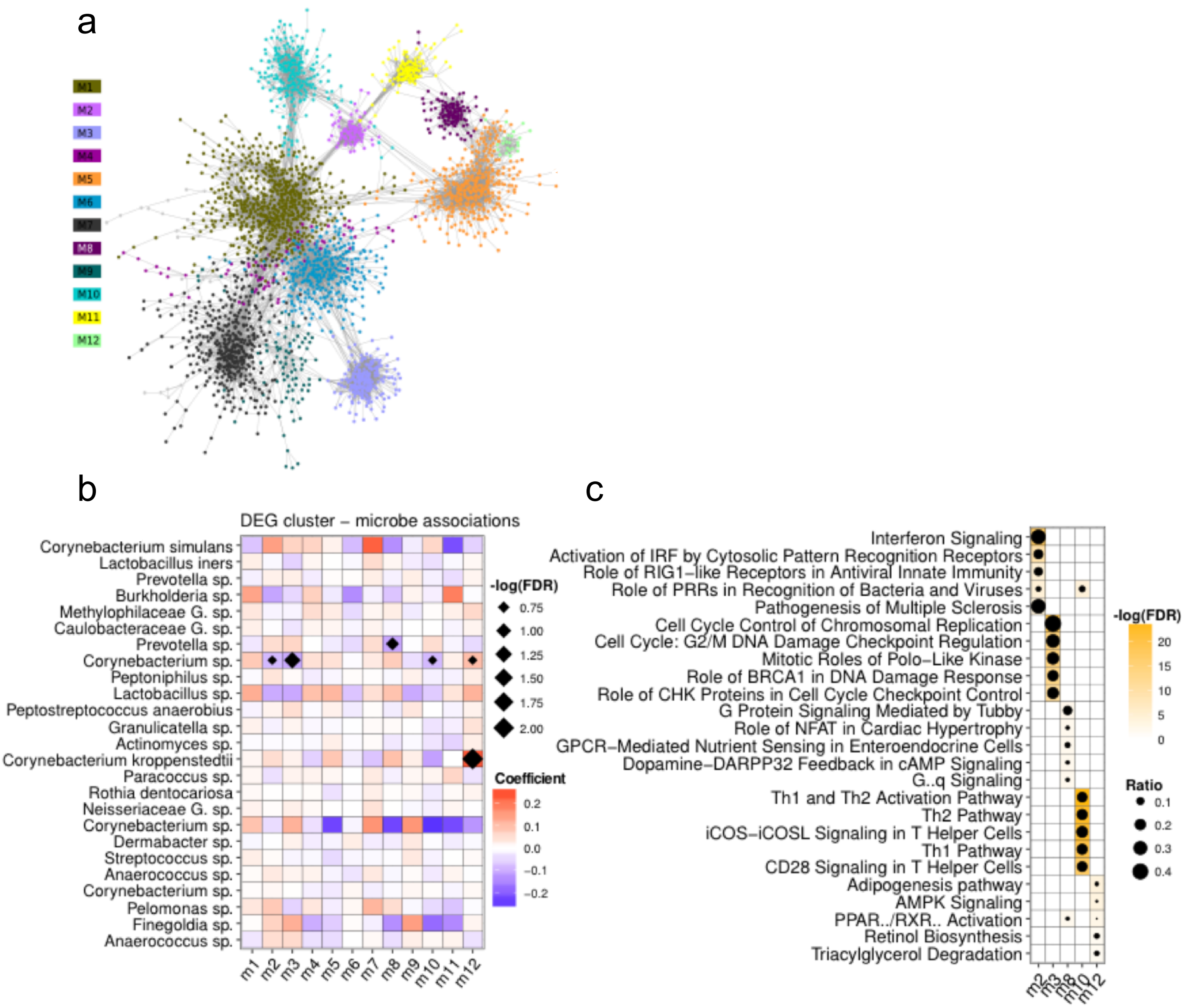

Supplementary Figure 14. Integrative analysis of PSO microbiome and transcriptome. a PSO co-expression network constructed from differentially expressed genes between HV (*n*=115) and PSO (*n*=119) samples resulting in a network of 2653 nodes. The network was partitioned into modules (coloured) using the Louvain method. **b** A linear model was fit for each microbe – module eigengene pair using MaAsLin. The color of heatmap squares correspond to the direction of association and squares correspond to the -log(FDR) of the association. Associations FDR <0.2 are shown. **c** Functional analysis of the microbial associated modules conducted using Ingenuity pathway analysis. The heatmap square corresponds to the significance of gene enrichment and the circle indicates the ratio of genes enriched to the total number of genes in the pathway. The source data files used to generate the present figure are available from the NCBI Sequence Read Archive under accession PRJNA554499, and from EBI ArrayExpress under accession E-MTAB-8149.

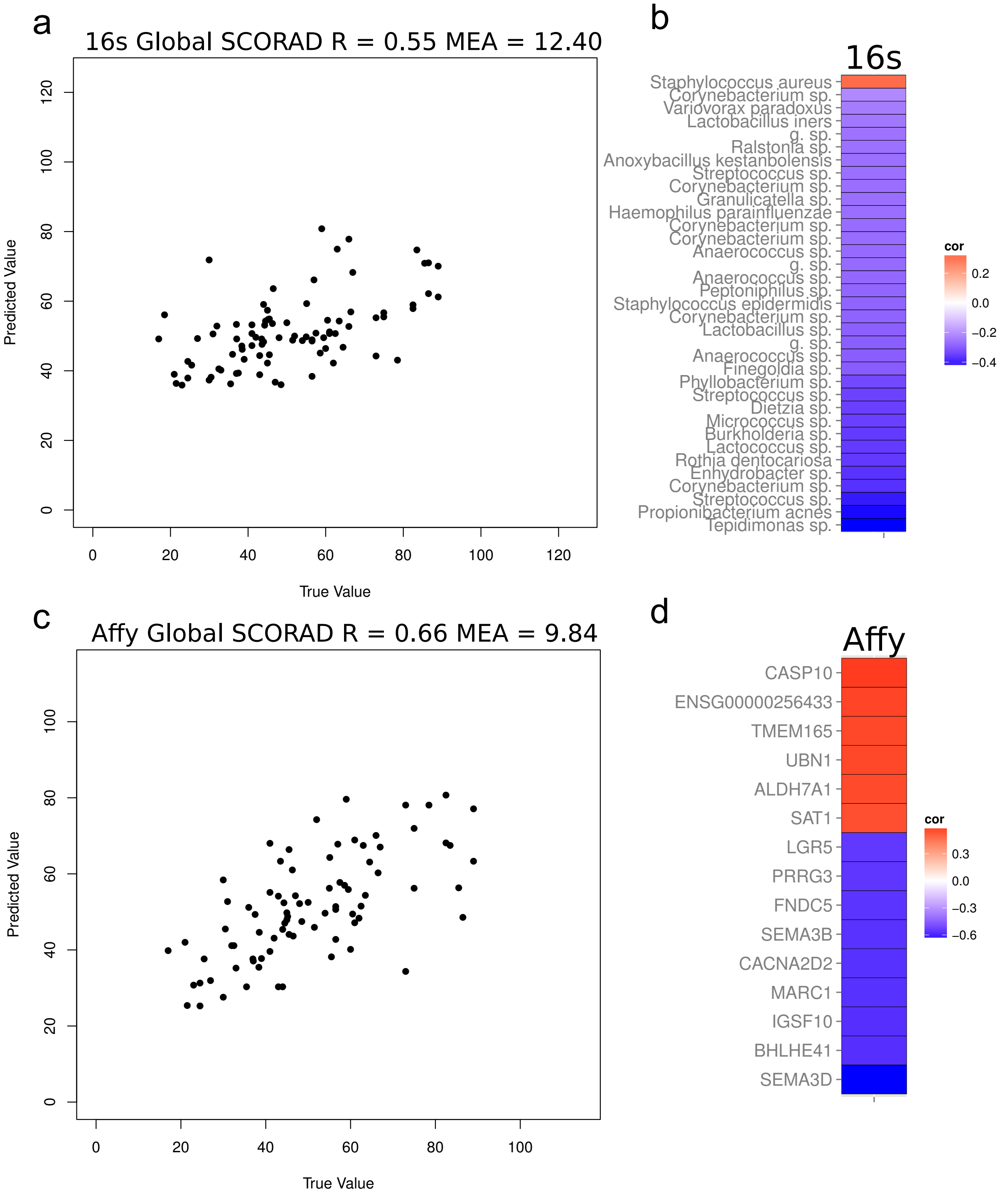

Supplementary Figure 15. Clinical severity and the AD associated microbiome and transcriptome. The models which achieved the lowest mean absolute error (MAE) are shown. **a** Predicted SCORAD from microbe abundance vs true SCORAD in AD (*n*=82). **b** The 35 selected OTUs included in the regression model. **c** Predicted SCORAD from gene expression vs true SCORAD. **d** The 15 selected genes included in the regression model. The source data files used to generate the present figure are available from the NCBI Sequence Read Archive under accession PRJNA554499, and from EBI ArrayExpress under accession E-MTAB-8149.

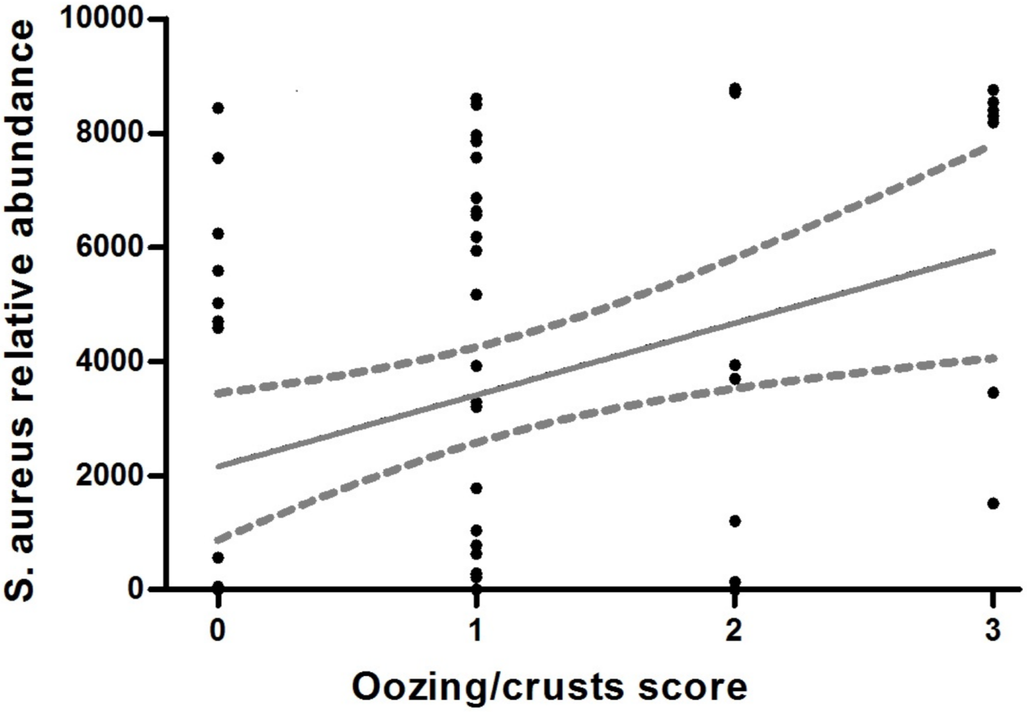

Supplementary Figure 16. Association between oozing and abundance of *S. aureus* in AD. Spearman rank-correlation of oozing/crusts clinical scoring and the relative abundance of *S. aureus*. Dashed lines indicate the 95% confidence interval (r=0.375, *p*=0.002). The source data files used to generate the present figure are available from the NCBI Sequence Read Archive under accession PRJNA554499, and from EBI ArrayExpress under accession E-MTAB-8149.

Supplementary Table 1. 16S rRNA gene sequencing results.

OTU	P-value	Statistics	Adjusted P-value	Lowest taxonomic rank
OTU883806	1.082796337616e-35	161.021862716733	1.0286565207352e-33	Staphylococcus aureus (s)
OTU912997	5.46283956660191e-20	88.7074662763379	2.59484879413591e-18	Corynebacterium simulans (s)
OTU837884	9.03605858513986e-12	50.8595960676359	2.86141855196096e-10	Burkholderia (g)
OTU1096610	2.5332545462453e-11	48.7978623299205	4.81318363786608e-10	Finegoldia (g)
OTU279980	2.47391663531714e-11	48.8452668916935	4.81318363786608e-10	Staphylococcus (g)
OTU360483	9.94086630171859e-11	46.0635637059465	1.57397049777211e-09	Neisseriaceae (f)
OTU4481323	1.25947011945663e-10	45.5903196750853	1.70928087640542e-09	Lactobacillus (g)
OTU819937	3.01928780229458e-10	43.8416599068401	3.58540426522482e-09	Caulobacteraceae (f)
OTU1107940	1.34892167706799e-09	40.8479206428008	1.42386177023844e-08	Pelomonas (g)
OTU403853	7.1974971193661e-09	37.4990649873386	6.8376222633978e-08	Propionibacterium acnes (s)
OTU4422718	8.11527430597365e-09	37.2590356684261	7.0086459915227e-08	Corynebacterium kroppenstedtii (s)
OTU2110555	2.62556441675415e-08	34.9107697102301	2.07857182993037e-07	Anaerococcus (g)
OTU164003	9.11608235073351e-08	32.4212811979238	6.66175248707449e-07	Bradyrhizobium (g)
OTU851668	3.21066791350797e-07	29.9032333254332	2.17866751273755e-06	Prevotella (g)
OTU285376	9.81901229364694e-07	27.6675502294912	6.21870778597639e-06	Corynebacterium (g)
OTU505749	1.27693599311721e-06	27.1420942100454	7.58180745913343e-06	Peptoniphilus (g)
OTU1131523	1.74038585381491e-06	26.5228074285116	9.72568565367155e-06	Corynebacterium (g)
OTU851925	2.17661576395271e-06	26.0754785784882	1.14876943097504e-05	Lactobacillus iners (s)
OTU370309	4.72918068278217e-06	24.5235171752701	2.36459034139108e-05	Anaerococcus (g)
OTU4047452	5.26026525989511e-06	24.3106582056967	2.39729623326655e-05	Kocuria palustris (s)
OTU4473664	5.29928641037868e-06	24.2958767720442	2.39729623326655e-05	Peptostreptococcus anaerobius (s)
OTU4349859	1.08143409972286e-05	22.8692748691204	4.66982906698509e-05	Enhydrobacter (g)
OTU4317476	1.25633952367307e-05	22.5694462241174	5.18922846734527e-05	Rothia dentocariosa (s)
OTU1003210	1.42751891537567e-05	22.313975103618	5.65059570669537e-05	Anaerococcus (g)
OTU565753	1.53247258273243e-05	22.1720859337037	5.82339581438323e-05	Corynebacterium (g)
OTU4439089	2.30010650514732e-05	21.3599400730429	8.40423530726907e-05	Actinomyces (g)
OTU25478	3.25462292613402e-05	20.6656980810517	0.000114514510363975	Dermabacter (g)
OTU820692	4.41632523074884e-05	20.0552350208799	0.000149839606043264	Methylophilaceae (f)
OTU2901965	8.18154204770622e-05	18.8220896354653	0.000268016032597273	Streptococcus (g)
OTU610043	8.72834319123579e-05	18.6926997929869	0.000276397534389133	Prevotella (g)
OTU4422405	0.000158343082584874	17.5014929405462	0.000485244930502033	Micrococcus (g)
OTU4467218	0.000188527688220535	17.1525313495029	0.000559691574404714	Ochrobactrum (g)
OTU4349522	0.000235861283115594	16.7045334183191	0.000678994602908529	Anaerococcus (g)
OTU4021335	0.000358285265261406	15.8683621170504	0.000972488577138102	Acinetobacter johnsonii (s)
OTU4327300	0.000354110582277029	15.8918026285942	0.000972488577138102	Anaerococcus (g)
OTU4474056	0.00037447852030314	15.7799522246355	0.000988207206355508	Peptoniphilus (g)
OTU4301457	0.000394243876987359	15.6770817260568	0.00101224779226484	Tepidimonas (g)
OTU25259	0.000442534685634347	15.4459834219479	0.00110633671408587	Streptococcus (g)
OTU761594	0.000509220645297227	15.1652582950573	0.00124040926418555	Thermoanaerobacterium (g)
OTU4482598	0.00067807366364214	14.5925092549719	0.00161042495115008	Acinetobacter (g)
OTU496787	0.00076154145347984	14.3603319009979	0.00176454727025817	Staphylococcus epidermidis (s)
OTU4348347	0.000801405338649813	14.2582873962601	0.0018006010078347	Paracoccus (g)
OTU4440643	0.000815008877230443	14.2246231049509	0.0018006010078347	Corynebacterium (g)
OTU4354809	0.00130177582451103	13.2880518556299	0.0027509488298228	Caulobacteraceae (f)
OTU4471315	0.00130308102465291	13.2860475992325	0.0027509488298228	Granulicatella (g)
OTU4327286	0.0015041503805283	12.9990541427989	0.00310639752500409	Corynebacterium (g)
OTU939571	0.00164066078125504	12.8253124057905	0.003316229238707	Anaerococcus (g)
OTU370134	0.00187793796083311	12.5551618668838	0.00371013416722575	Blautia (g)
OTU4473201	0.00191364814941118	12.5174876656678	0.00371013416722575	Sphingomonadaceae (f)
OTU4460228	0.00233330185332872	12.120941820233	0.00443327352132457	Corynebacterium (g)
OTU1081372	0.00632547412707488	10.1263405727512	0.011556154655233	WAL_1855D (g)
OTU20360	0.00629722458427567	10.1352925696607	0.011556154655233	Corynebacterium (g)
OTU114999	0.00731884202030772	9.83460631493986	0.0131186790930044	Corynebacterium (g)
OTU654307	0.00769845054354239	9.73347239696594	0.0135435704006764	Peptoniphilus (g)
OTU912906	0.00948918001575288	9.31520615035486	0.0163904018453913	Anaerococcus (g)
OTU851917	0.011185686631957	8.98624059456517	0.0189757183934985	Prevotella (g)
OTU625320	0.0132115829154091	8.65332268114129	0.0220193048590151	Peptoniphilus (g)
OTU13445	0.0134685490608132	8.6147960214163	0.0220605544961595	Corynebacterium (g)
OTU1004369	0.0139576671582279	8.54345260924328	0.0220996396671941	Streptococcus (g)
OTU4449324	0.0137807034987041	8.56897192508829	0.0220996396671941	Cloacibacterium (g)
OTU4306540	0.0151226364554817	8.38312510936484	0.023551646938865	Acinetobacter (g)
OTU247720	0.0167547193535348	8.17815061589198	0.0256725538481582	Brevibacterium paucivorans (s)
OTU995817	0.0187009737330081	7.95835937038681	0.0281998810259646	Corynebacterium (g)
OTU755148	0.0199358610373192	7.83047021368167	0.0291370276699281	Jan-68 (g)
OTU987144	0.0196880552466783	7.8554863195762	0.0291370276699281	Corynebacterium (g)
OTU4456068	0.0290090109132525	7.08029755222569	0.0417553944963483	Variovorax paradoxus (s)
OTU378096	0.0328340217314866	6.83258010650156	0.046555702455093	Acinetobacter (g)
OTU74351	0.0357143826595617	6.66440359142232	0.0498950934214464	Streptophyta (o)
OTU4294554	0.0400515715065258	6.43517473524541	0.0551434680162312	Facklamia (g)
OTU4318084	0.0409429245853392	6.39115253082071	0.0555653976515317	Gemellaceae (f)
OTU4476950	0.0419542718473155	6.34235003874928	0.0561359975421827	Anaerococcus (g)
OTU4346894	0.0524125952794498	5.8972166972209	0.0691555076603852	Phyllobacterium (g)
OTU3208510	0.0632800781337968	5.5203694415844	0.0823507866124753	Brachybacterium conglomeratum (s)
OTU14278	0.071302751129314	5.28164073408482	0.0915373156389842	Peptoniphilus (g)
OTU4303697	0.0743916983036289	5.19682185061858	0.0942294845179299	Acinetobacter (g)
OTU4353642	0.0786584697969721	5.08527993109938	0.0983230872462151	Paracoccus aminovorans (s)
OTU4480063	0.0853966582885274	4.92089661812827	0.105359513472859	Ralstonia (g)
OTU103810	0.0939155470503113	4.73071867236463	0.114384320125379	Janibacter (g)
OTU3841245	0.113935116931669	4.34425228467405	0.137010583652008	Corynebacterium (g)
OTU4421536	0.119113987340656	4.25534873505924	0.141447859967029	Aerococcus (g)
OTU4408996	0.126264243391992	4.13875679648756	0.148087692867151	Anoxybacillus kestanbolensis (s)
OTU4349519	0.127886327306842	4.1132269550875	0.148160988953048	Anaerococcus (g)
OTU4350124	0.155484883033482	3.72241353461464	0.177964625158805	Acinetobacter (g)
OTU282360	0.187876566645355	3.3439401843588	0.212479450372723	Corynebacterium (g)
OTU940083	0.201340613631936	3.20551441989136	0.225027744647458	Corynebacterium (g)
OTU511475	0.217936652034388	3.0471016908397	0.240743976084498	Streptococcus (g)
OTU4369229	0.220543954300249	3.02331652946101	0.24082385814395	Burkholderiales (o)
OTU1036883	0.275744867799313	2.57655846562051	0.297679118646985	Kocuria rhizophila (s)
OTU4468125	0.340367969441369	2.15545596706709	0.363314124684607	Rhodococcus (g)
OTU205025	0.416205665397651	1.75315150583044	0.439328202364188	Chryseobacterium (g)
OTU4411187	0.428524598501511	1.69481427304153	0.447360844589489	Dietzia (g)
OTU4309323	0.449932623851577	1.59731486440384	0.464604339846737	Haemophilus parainfluenzae (s)
OTU4446521	0.567899838188449	1.13162043405689	0.580112737934437	Lactococcus (g)
OTU362390	0.77102569799755	0.520067150476283	0.779228099040078	Corynebacterium (g)
OTU4299324	0.928857522610018	0.147599836669691	0.928857522610018	Streptococcus (g)

(s)=species, (g)=genus, (f)=family, (o)=order

Statistical analysis (Kruskal-Wallis test, FDR, $p<0.05$) of the 16S rRNA gene sequencing results. The source data files used to generate the present table are available from the NCBI Sequence Read Archive under accession PRJNA554499.

Supplementary Table 2. Results of the confounder analysis.

Table for AD

OTU	*P*-value	confounders	Adjusted *P*-value	taxname
**OTU1096610**	0.000495857	Institution;anatomical_location	0.001685915	*Finegoldia* spp.
**OTU13445**	0.184305984	CUSTOM_Age	0.241015517	*Corynebacterium* spp.
**OTU164003**	1.28E-05	Institution	0.000108666	*Bradyrhizobium* spp.
**OTU378096**	0.775397085	Institution	0.823859403	*Acinetobacter* spp.
**OTU4021335**	0.855576102	Institution	0.855576102	*A.johnsonii*
**OTU4349522**	9.19E-05	Institution	0.000496613	*Anaerococcus* spp.
**OTU4354809**	0.212677095	Institution	0.258250758	*Caulobacteraceae*
**OTU4422405**	0.009189874	Institution	0.019528481	*Micrococcus* spp.
**OTU4456068**	0.00011685	Institution;anatomical_location	0.000496613	*V. paradoxus*
**OTU4481323**	0.001519295	Gender	0.003689717	*Lactobacillus* spp.
**OTU4482598**	0.630874065	Institution	0.714990607	*Acinetobacter* spp.
**OTU820692**	0.03989263	Institution	0.075352746	*Methylophilaceae*
**OTU851668**	0.069680293	Institution	0.107687725	*Prevotella* sp.
**OTU851925**	0.099164356	Gender;Institution;CUSTOM_Age	0.140482837	*L. iners*
**OTU883806**	2.65E-37	anatomical_location	4.50E-36	*S. aureus*
**OTU912906**	0.05409198	Gender	0.091956366	*Anaerococcus* spp.
**OTU939571**	0.00103751	Institution	0.002939612	*Anaerococcus* spp.

Table for PSO

OTU	*P*-value	confounders	Adjusted *P*-value	taxname
**OTU1096610**	0.000198593	Institution;anatomical_location	0.001290854	*Finegoldia* spp.
**OTU164003**	0.00260733	Institution	0.008473823	*Bradyrhizobium* spp.
**OTU285376**	6.77E-05	anatomical_location	0.000880102	*Corynebacterium* spp.
**OTU4327286**	0.756887546	Gender	0.756887546	*Corynebacterium* spp.
**OTU4354809**	0.335223099	Institution	0.396172753	*Caulobacteraceae*
**OTU4422405**	0.16060262	Institution	0.231981562	*Micrococcus* spp.
**OTU4422718**	0.000600743	CUSTOM_Age	0.002603218	*C. kroppenstedtii*
**OTU4439089**	0.011351491	Institution	0.029513877	*Actinomyces* spp.
**OTU4460228**	0.204028455	Gender	0.265236991	*Corynebacterium* spp.
**OTU4481323**	0.031441572	Gender	0.068123407	*Lactobacillus* spp.
**OTU820692**	0.092479113	Institution	0.158389881	*Methylophilaceae*
**OTU851668**	0.097470696	Institution	0.158389881	*Prevotella* spp.
**OTU851925**	0.579111601	Gender;Institution;CUSTOM_Age	0.627370901	*L. iners*

The table shows the *p*-values and adjusted *p*-values after correction for confounding factors, for abundant OTUs that were significantly associated with disease and also significantly associated with a confounded effect. Associations were tested using the Kruskal-Wallis test for body site and institution, the Mann Whitney U test for gender, and Spearman correlation for age. OTUs with an adjusted *p*-value lower than 0.01 retain significance after correction. The source data files used to generate the present tables are available from the NCBI Sequence Read Archive under accession PRJNA554499.

Supplementary Table 3. The *S. aureus* signature.

ID	Symbol	Expr Log Ratio	Expr p-value
ENSG00000184330	S100A7A	2,851	0,0106
ENSG00000244094	SPRR2F	2,65	0,00643
ENSG00000171711	DEFB4A/DEFB4B	2,601	0,0126
ENSG00000124102	PI3	2,013	0,0223
ENSG00000153802	TMPRSS11D	1,652	0,00643
ENSG00000262406	MMP12	1,484	0,0171
ENSG00000163220	S100A9	1,472	0,0248
ENSG00000181333	HEPHL1	1,454	0,038
ENSG00000138135	CH25H	1,358	0,0077
ENSG00000101670	LIPG	1,344	0,024
ENSG00000007908	SELE	1,289	0,0283
ENSG00000104368	PLAT	1,257	0,0183
ENSG00000174502	SLC26A9	1,234	0,0184
ENSG00000064886	CHI3L2	1,174	0,0153
ENSG00000268104	SLC6A14	1,162	0,0185
ENSG00000248329	APELA	1,123	0,0243
ENSG00000165474	GJB2	1,087	0,016
ENSG00000231027		1,081	0,00643
ENSG00000151006	PRSS53	1,06	0,0418
ENSG00000062096	ARSF	1,053	0,0107
ENSG00000137648	TMPRSS4	1,018	0,0357
ENSG00000167772	ANGPTL4	1,014	0,0245
ENSG00000104972	LILRB1	0,982	0,00643
ENSG00000157551	KCNJ15	0,959	0,019
ENSG00000108691	CCL2	0,947	0,0106
ENSG00000115919	KYNU	0,942	0,0297
ENSG00000113749	HRH2	0,929	0,0202
ENSG00000206073	SERPINB4	0,927	0,043
ENSG00000169684	CHRNA5	0,915	0,00918
ENSG00000146574	CCZ1/CCZ1B	0,913	0,0185
ENSG00000091137	SLC26A4	0,894	0,017
ENSG00000227300	KRT16P2	0,887	0,0275
ENSG00000158125	XDH	0,885	0,0119
ENSG00000236481	LINC02195	0,876	0,0463
ENSG00000057149	SERPINB3	0,871	0,0185
ENSG00000151790	TDO2	0,857	0,00688
ENSG00000173559	NABP1	0,83	0,0323
ENSG00000279277		0,824	0,0208
ENSG00000163638	ADAMTS9	0,817	0,0202
ENSG00000143546	S100A8	0,814	0,0248
ENSG00000172724	CCL19	0,807	0,0126
ENSG00000149573	MPZL2	0,773	0,023
ENSG00000225489		0,772	0,0294
ENSG00000171208	NETO2	0,765	0,039
ENSG00000164687	FABP5	0,763	0,0237
ENSG00000211964	IGHV3-48	0,763	0,0223
ENSG00000135074	ADAM19	0,757	0,0482
ENSG00000070501	POLB	0,755	0,0118
ENSG00000170542	SERPINB9	0,75	0,0236
ENSG00000157193	LRP8	0,743	0,0144
ENSG00000184254	ALDH1A3	0,739	0,0262
ENSG00000062038	CDH3	0,738	0,0353
ENSG00000268043	NBPF10	0,737	0,0397
ENSG00000164430	CGAS	0,735	0,011
ENSG00000271856	LINC01215	0,723	0,0159
ENSG00000189057	FAM111B	0,722	0,028
ENSG00000143839	REN	0,713	0,0353
ENSG00000163874	ZC3H12A	0,709	0,00832
ENSG00000025708	TYMP	0,707	0,0352
ENSG00000019186	CYP24A1	0,7	0,0299
ENSG00000232445		0,691	0,0416
ENSG00000111801	BTN3A3	0,673	0,0135
ENSG00000256433		0,668	0,0151
ENSG00000062716	VMP1	0,661	0,0314
ENSG00000105835	NAMPT	0,655	0,016
ENSG00000117009	KMO	0,653	0,0208
ENSG00000148459	PDSS1	0,65	0,0248
ENSG00000155380	SLC16A1	0,648	0,0385
ENSG00000251402	FAM90A25P	0,641	0,0119
ENSG00000184731	FAM110C	0,633	0,0474
ENSG00000188681	TEKT4P2	0,633	0,0314
ENSG00000103522	IL21R	0,625	0,0181
ENSG00000105639	JAK3	0,624	0,0171
ENSG00000077238	IL4R	0,622	0,04
ENSG00000145287	PLAC8	0,62	0,0184
ENSG00000119714	GPR68	0,616	0,0245
ENSG00000030110	BAK1	0,615	0,0106
ENSG00000097046	CDC7	0,614	0,0219
ENSG00000160766	GBAP1	0,605	0,0467
ENSG00000151014	NOCT	0,601	0,0457
ENSG00000021355	SERPINB1	0,598	0,0445
ENSG00000119630	PGF	0,593	0,00806
ENSG00000042062	RIPOR3	0,591	0,0159
ENSG00000196584	XRCC2	0,587	0,011
ENSG00000258689	LINC01269	0,585	0,0495
ENSG00000155189	AGPAT5	0,584	0,0243
ENSG00000104213	PDGFRL	-0,583	0,0243
ENSG00000151623	NR3C2	-0,584	0,017
ENSG00000160862	AZGP1	-0,587	0,0181
ENSG00000108231	LGI1	-0,591	0,027
ENSG00000113083	LOX	-0,591	0,0462
ENSG00000253047		-0,592	0,0446
ENSG00000112796	ENPP5	-0,592	0,04
ENSG00000151892	GFRA1	-0,597	0,0213
ENSG00000268460	LOC93429	-0,597	0,0284
ENSG00000166770	ZNF667-AS1	-0,597	0,0237
ENSG00000220483	SLC25A51P1	-0,599	0,0265
ENSG00000163331	DAPL1	-0,6	0,028
ENSG00000168405	CMAHP	-0,603	0,042
ENSG00000173926	MARCH3	-0,605	0,00917
ENSG00000076716	GPC4	-0,606	0,019
ENSG00000007306	CEACAM7	-0,607	0,0287
ENSG00000189169	KRTAP10-12	-0,607	0,0482
ENSG00000092607	TBX15	-0,609	0,0101
ENSG00000257524		-0,61	0,0444
ENSG00000143867	OSR1	-0,61	0,0187
ENSG00000187372	PCDHB13	-0,614	0,0188
ENSG00000163710	PCOLCE2	-0,614	0,0298
ENSG00000168952	STXBP6	-0,614	0,0139
ENSG00000164920	OSR2	-0,615	0,0263
ENSG00000122707	RECK	-0,619	0,0219
ENSG00000170549	IRX1	-0,622	0,0213
ENSG00000196734	LCE1B	-0,627	0,0277
ENSG00000184347	SLIT3	-0,628	0,00894
ENSG00000070388	FGF22	-0,629	0,0187
ENSG00000106078	COBL	-0,635	0,0202
ENSG00000170370	EMX2	-0,639	0,0181
ENSG00000103319	EEF2K	-0,643	0,0353
ENSG00000095539	SEMA4G	-0,643	0,0398
ENSG00000201134		-0,65	0,00275
ENSG00000174514	MFSD4A	-0,653	0,0349
ENSG00000138669	PRKG2	-0,654	0,0428
ENSG00000070601	FRMPD1	-0,66	0,0183
ENSG00000188624	IGFL3	-0,661	0,0405
ENSG00000165124	SVEP1	-0,661	0,0388
ENSG00000198816	ZNF358	-0,666	0,00275
ENSG00000187173	LCE2A	-0,668	0,0214
ENSG00000131831	RAI2	-0,668	0,00643
ENSG00000231604		-0,669	0,0187
ENSG00000133083	DCLK1	-0,671	0,0147
ENSG00000168447	SCNN1B	-0,673	0,00808
ENSG00000100234	TIMP3	-0,674	0,00105
ENSG00000135063	FAM189A2	-0,676	0,0188
ENSG00000148671	ADIRF	-0,678	0,017
ENSG00000227051	C14orf132	-0,678	0,00688
ENSG00000006747	SCIN	-0,678	0,0272
ENSG00000182898	TCHHL1	-0,681	0,0245
ENSG00000197953	AADACL2	-0,682	0,00564
ENSG00000176971	FIBIN	-0,682	0,0314
ENSG00000229255		-0,685	0,0336
ENSG00000198542	ITGBL1	-0,687	0,0154
ENSG00000251580	LINC02482	-0,687	0,028
ENSG00000185345	PRKN	-0,69	0,0265
ENSG00000136155	SCEL	-0,69	0,0265
ENSG00000115112	TFCP2L1	-0,692	0,00808
ENSG00000254835	RNF185-AS1	-0,694	0,0259
ENSG00000157514	TSC22D3	-0,695	0,00643
ENSG00000189320	FAM180A	-0,7	0,00643
ENSG00000125845	BMP2	-0,701	0,0398
ENSG00000204789	ZNF204P	-0,701	0,0192
ENSG00000203786	KPRP	-0,704	0,019
ENSG00000198854	C1orf68	-0,705	0,0229
ENSG00000188783	PRELP	-0,707	0,011
ENSG00000185940	KRTAP5-3/KRTAP5-5	-0,712	0,0495
ENSG00000184564	SLITRK6	-0,712	0,0282
ENSG00000122674	CCZ1/CCZ1B	-0,713	0,0277
ENSG00000125355	TMEM255A	-0,716	0,0337
ENSG00000168477	TNXB	-0,719	0,00653
ENSG00000170962	PDGFD	-0,72	0,0181
ENSG00000239521	GATS	-0,723	0,00643
ENSG00000248290	TNXA	-0,726	0,0424
ENSG00000000005	TNMD	-0,728	0,0243
ENSG00000201608	RNU4-42P	-0,729	0,0246
ENSG00000152377	SPOCK1	-0,732	0,0146
ENSG00000169252	ADRB2	-0,733	0,0104
ENSG00000186207	LCE5A	-0,736	0,00841
ENSG00000232220		-0,739	0,0184
ENSG00000183287	CCBE1	-0,739	0,0213
ENSG00000184022	OR2T10	-0,739	0,0314
ENSG00000102934	PLLP	-0,741	0,0183
ENSG00000114771	AADAC	-0,743	0,0126
ENSG00000189014	FAM35DP	-0,744	0,0487

For identification of differentially expressed genes between *S. aureus* 'high' and 'low' samples, a linear model (R package Limma) was fitted to the data, and pairwise comparisons were done using the empirical Bayes method. Transcriptomes were defined based on a fold change of 1.5 or greater and a Benjamini-Hochberg adjusted *p*-value less than 0.05. The source data files used to generate the present table are available from the NCBI Sequence Read Archive under accession PRJNA554499, and from EBI ArrayExpress under accession E-MTAB-8149.

Supplementary Table 3, continued…

ID	Symbol	Expr Log Ratio	Expr p-value
ENSG00000205221	VIT	-0,745	0,0104
ENSG00000105143	SLC1A6	-0,746	0,00643
ENSG00000170381	SEMA3E	-0,748	0,0181
ENSG00000172201	ID4	-0,751	0,0304
ENSG00000176387	HSD11B2	-0,754	0,0168
ENSG00000214783	POLR2J4	-0,754	0,00643
ENSG00000203392		-0,758	0,0193
ENSG00000119138	KLF9	-0,761	0,00841
ENSG00000178776	C5orf46	-0,771	0,0276
ENSG00000187170	LCE4A	-0,774	0,0251
ENSG00000106123	EPHB6	-0,777	0,0145
ENSG00000143412	ANXA9	-0,778	0,0387
ENSG00000249731		-0,78	0,0402
ENSG00000165953	SERPINA12	-0,786	0,0438
ENSG00000259225	LINC02345	-0,789	0,00887
ENSG00000144891	AGTR1	-0,791	0,0118
ENSG00000234477		-0,796	0,00688
ENSG00000185950	IRS2	-0,797	0,00643
ENSG00000153930	ANKFN1	-0,8	0,0306
ENSG00000111341	MGP	-0,8	0,00311
ENSG00000179954	SSC5D	-0,805	0,00678
ENSG00000212658	KRTAP29-1	-0,835	0,0448
ENSG00000261609	GAN	-0,84	0,0331
ENSG00000205864	KRTAP5-6	-0,844	0,0304
ENSG00000153993	SEMA3D	-0,846	0,00982
ENSG00000163145	C1QTNF7	-0,853	0,00345
ENSG00000163083	INHBB	-0,853	0,00275
ENSG00000166415	WDR72	-0,859	0,0341
ENSG00000187223	LCE2C/LCE2D	-0,86	0,0243
ENSG00000197614	MFAP5	-0,866	0,00841
ENSG00000221305	mir-548	-0,871	0,0349
ENSG00000137809	ITGA11	-0,881	0,00643
ENSG00000212900	KRTAP3-2	-0,883	0,0149
ENSG00000235942	LCE6A	-0,883	0,023
ENSG00000244411	KRTAP5-7	-0,901	0,0287
ENSG00000186393	KRT26	-0,919	0,0261
ENSG00000196859	KRT39	-0,919	0,0405
ENSG00000269729		-0,928	0,0324
ENSG00000143520	FLG2	-0,933	0,0192
ENSG00000127083	OMD	-0,943	0,0185
ENSG00000116678	LEPR	-0,951	0,0337
ENSG00000156284	CLDN8	-0,967	0,0288
ENSG00000186049	KRT73	-0,975	0,028
ENSG00000279301	OR2T11	-0,975	0,0243
ENSG00000170615	SLC26A5	-0,977	0,00366
ENSG00000197594	ENPP1	-0,982	0,00345
ENSG00000131686	CA6	-0,988	0,023
ENSG00000170486	KRT72	-0,992	0,039
ENSG00000116039	ATP6V1B1	-0,996	0,0139
ENSG00000197084	LCE1C	-1,012	0,00643
ENSG00000187151	ANGPTL5	-1,016	0,023
ENSG00000139292	LGR5	-1,021	0,046
ENSG00000165072	MAMDC2	-1,025	0,00678
ENSG00000102683	SGCG	-1,042	0,00345
ENSG00000204572	KRTAP5-10	-1,046	0,0183
ENSG00000119457	SLC46A2	-1,051	0,0118
ENSG00000109906	ZBTB16	-1,055	0,00808
ENSG00000250641	LY6G6F-LY6G6D	-1,061	0,0392
ENSG00000149090	PAMR1	-1,092	0,00643
ENSG00000196616	ADH1B	-1,127	0,0083
ENSG00000168079	SCARA5	-1,135	0,00638
ENSG00000162040	HS3ST6	-1,148	0,0355
ENSG00000166869	CHP2	-1,161	0,0248
ENSG00000124939	SCGB2A1	-1,166	0,0146
ENSG00000179094	PER1	-1,189	0,00841
ENSG00000232324		-1,218	0,000241
ENSG00000159455	LCE2B	-1,241	0,00643
ENSG00000226005		-1,243	0,0199
ENSG00000111291	GPRC5D	-1,25	0,0456
ENSG00000125571	IL37	-1,257	0,0406
ENSG00000116194	ANGPTL1	-1,285	0,00643
ENSG00000212725	KRTAP2-1	-1,306	0,028
ENSG00000094796	KRT31	-1,326	0,0388
ENSG00000204571	KRTAP5-11	-1,407	0,028
ENSG00000188694	KRTAP24-1	-1,438	0,0498
ENSG00000161850	KRT82	-1,447	0,0413
ENSG00000186860	KRTAP17-1	-1,537	0,0275
ENSG00000196224	KRTAP5-3/KRTAP5-5	-1,787	0,0377
ENSG00000241598	KRTAP5-4	-1,8	0,0202
ENSG00000159763	PIP	-1,922	0,0341
ENSG00000214518	KRTAP2-2	-2,052	0,0326
ENSG00000212657	KRTAP16-1	-2,067	0,029
ENSG00000205867	KRTAP5-2	-2,277	0,0304
ENSG00000241233	KRTAP5-8	-2,422	0,0189

Supplementary Table 4. Tryptophan dependence of AD associated *S. aureus* strains.

S. aureus strains	Trp dependence NO	YES
Donor #1		x
Donor #2		x
Donor #3		x
Donor #4	x	
Donor #5	x	
Donor #6		x
Donor #7	x	
Donor #8	x	
Donor #9	x	
Donor #10	x	
Donor #11		x
Donor #12	x	
Donor #13		x
Donor #14	x	
Donor #15	x	
Donor #16	x	
Donor #17	x	
Donor #18	x	
Donor #19	x	
Donor #20	x	
Donor #21	x	
Donor #22	x	
Donor #23	x	
Donor #24		x
Donor #25		x
Donor #26	x	
Donor #27		x
Donor #28	x	
Donor #29	x	
Donor #30	x	
Donor #31		x
Donor #32		x
Σ	66% (21/32)	34% (11/32)

Supplementary Table 5. Primers used in the study.

Primers used in the study						
**Gene expression analysis of 3d human epidermal equivalents (HEEs)**						
Hugo Gene Symbol	Forward primer 5'>3'	Reverse primer 5'>3'	cDNA specific	Slope	E fold	E %
DEFB4	gatgcctccttccaggtgtttt	ggatgacatatggctccactctt	yes	-3.36	1.99	99.3
PI3	catgagggccagcagctt	tttaacaggaactcccgtgaca	yes	-3.3	2.02	100.9
IL4R	ctgcctgttgtgctatgtc	tctgatcccaccattctttct	yes	-3.16	2.1	105.2
S100A8	ccgagtgtcctcagtatatcaggaa	acgcccatctttatcaccagaat	yes	-3.36	1.98	99.2
**16S rRNA gene sequencing**						
Forward primer	Sequence	Adaptors	Reverse primer	Sequence	Adaptors	
341f	5′-CCTACGGGNGGCWGCAG	adptor B, Lib-L	805r	5′-GACTACHVGGGTATCTAATCC	adaptor A, Lib-L	
