# Supplementary material for: Microbe-host interplay in atopic dermatitis and psoriasis
Source: Nat Commun. 2019 Oct 16;10:4703. doi: 10.1038/s41467-019-12253-y (PMC6795799; doi:10.1038/s41467-019-12253-y)
Supplement: Supplementary file 3 — Reporting Summary [file 41467_2019_12253_MOESM3_ESM.pdf]

nature research

Corresponding author(s): Harri Alenius

Last updated by author(s): Aug 26, 2019

Reporting Summary

Nature Research wishes to improve the reproducibility of the work that we publish. This form provides structure for consistency and transparency in reporting. For further information on Nature Research policies, see Authors & Referees and the Editorial Policy Checklist.

Please do not complete any field with "not applicable" or n/a. Refer to the help text for what text to use if an item is not relevant to your study. For final submission: please carefully check your responses for accuracy; you will not be able to make changes later.

Statistics

For all statistical analyses, confirm that the following items are present in the figure legend, table legend, main text, or Methods section.

n/a	Confirmed	
☐	☒	The exact sample size (*n*) for each experimental group/condition, given as a discrete number and unit of measurement
☐	☒	A statement on whether measurements were taken from distinct samples or whether the same sample was measured repeatedly
☐	☒	The statistical test(s) used AND whether they are one- or two-sided<br>*Only common tests should be described solely by name; describe more complex techniques in the Methods section.*
☐	☒	A description of all covariates tested
☐	☒	A description of any assumptions or corrections, such as tests of normality and adjustment for multiple comparisons
☐	☒	A full description of the statistical parameters including central tendency (e.g. means) or other basic estimates (e.g. regression coefficient) AND variation (e.g. standard deviation) or associated estimates of uncertainty (e.g. confidence intervals)
☐	☒	For null hypothesis testing, the test statistic (e.g. *F*, *t*, *r*) with confidence intervals, effect sizes, degrees of freedom and *P* value noted<br>*Give P values as exact values whenever suitable.*
☐	☐	For Bayesian analysis, information on the choice of priors and Markov chain Monte Carlo settings
☐	☒	For hierarchical and complex designs, identification of the appropriate level for tests and full reporting of outcomes
☐	☒	Estimates of effect sizes (e.g. Cohen's *d*, Pearson's *r*), indicating how they were calculated

Our web collection on statistics for biologists contains articles on many of the points above.

Software and code

Policy information about availability of computer code

Data collection	No software used
Data analysis	The computational analyses presented in the manuscript did not utilize novel software or custom code that was central to the conclusions. The presented analyses may be accurately replicated using software tools and parameters described in Materials and Methods

For manuscripts utilizing custom algorithms or software that are central to the research but not yet described in published literature, software must be made available to editors/reviewers. We strongly encourage code deposition in a community repository (e.g. GitHub). See the Nature Research guidelines for submitting code & software for further information.

Data

Policy information about availability of data

All manuscripts must include a data availability statement. This statement should provide the following information, where applicable:

- Accession codes, unique identifiers, or web links for publicly available datasets
- A list of figures that have associated raw data
- A description of any restrictions on data availability

Data underlying Fig.s 1,2,3,4, Supplementary Fig.s 1,2,3,4,5,6,7,8,9,10,11,12,13,14,15,16 and Supplementary Tables 1,2,3,4 are provided as Source Data files. All other data are available from the corresponding author upon reasonable requests.

Field-specific reporting

Please select the one below that is the best fit for your research. If you are not sure, read the appropriate sections before making your selection.

☒ Life sciences ☐ Behavioural & social sciences ☐ Ecological, evolutionary & environmental sciences

Life sciences study design

All studies must disclose on these points even when the disclosure is negative.

Sample size	For sufficient sample size, a model based on 90% power to detect 2-fold changes at a significance level of 1% (Bonferroni adjusted) suggested group sizes of n=25 were required to achieve this for single factor analysis. For modeling data across several factors (clinical data, signaling pathways, microbiota), 80-100 samples would be sufficient according to previous experience. Therefore, n= 80-120 per group was estimated as sufficiently powered. In case we would fall short of samples for specific pathways or taxa, we would use statistical tests (students t test, ANOVA) to estimate false discovery rates (FDR), false negative rates (FNR), power, alpha and beta risks, which enable us to define the optimal sample size for maximized power and minimized FDR and FNR for multiple comparisons.
Data exclusions	A quality control pipeline based on the array Quality Metrics method was used to capture quality failures in microarray data, and based on these, a total of 12 samples were removed from the data set. Regarding the microbiome data, only OTUs present in more than 25% of alla samples were analysed. To reduce false positives in the contamination analysis, OTUs were discarded if they were present in less than 5 samples or if the raw abundance of the OTU in any sample was less than 10 reads.
Replication	All findings were replicated. Analysis of the large data set (AD=82, PSO=119, HV=115) for the microbiome and transcriptomics data, was preceded by a pilot analysis of 15 AD samples, 15 PSO samples and 15 HV samples, which showed similar results.
Randomization	AD patients with chronic atopic dermatitis, or PSO patients with plaque-type psoriasis were recruited to the study at random. Covariates such as secondary disease (eg. concomitant autoimmune diseases), were controlled for by adhering to specific exclusion criteria. Clinical information such as sex, age, onset of disease, etc were collected, and care was taken to recruit age and gender matched healthy volunteers.
Blinding	To identify gene or taxonomical markers that can predict disease severity, we used trained regression models, to which unseen samples were made blind, and used to estimate the accuracy of the trained model.

Reporting for specific materials, systems and methods

We require information from authors about some types of materials, experimental systems and methods used in many studies. Here, indicate whether each material, system or method listed is relevant to your study. If you are not sure if a list item applies to your research, read the appropriate section before selecting a response.

Materials & experimental systems

n/a	Involved in the study	
☒	☐	Antibodies
☒	☐	Eukaryotic cell lines
☒	☐	Palaeontology
☒	☐	Animals and other organisms
☐	☒	Human research participants
☐	☒	Clinical data

Methods

n/a	Involved in the study	
☒	☐	ChIP-seq
☒	☐	Flow cytometry
☒	☐	MRI-based neuroimaging

Human research participants

Policy information about studies involving human research participants

Population characteristics	AD patients with chronic atopic dermatitis, or PSO patients with plaque-type psoriasis were recruited to the study at random. Covariates such as secondary disease (eg. concomitant autoimmune disease), were controlled for by adhering to specific exclusion criteria. Moreover, exclusion criteria included the use of antibiotics within 2 weeks and systemic immunsuppressive theray or phototherapy or systemic biologic agents within the previous 12 weeks prior to screening. Clinical information such as sex, age, onset of disease, family anamnesis, allergies, positive allergen tests, total IgE levels, specific IgE, drug anamnesis and disease anamnesis were collected. Care was taken to recruit age and gender matched healthy volunteers.
Recruitment	The recruitment of subjects to the study was made based on physical examination by a dermatologist, and the resulting diagnosis. Patients or healthy volunteers that did not match inclusion/exclusion criteria were removed from the study.
Ethics oversight	The study was approve by the appropriate local Institutional Review Boards at University of Helsinki (Dnro 91/13/03/00/2011), Heinrich Heine University Duesseldorf (3647/2011) and King's College London (11/H0802/6). All subjects provided written

	informed consent before participation.

Note that full information on the approval of the study protocol must also be provided in the manuscript.

Clinical data

Policy information about clinical studies

All manuscripts should comply with the ICMJE guidelines for publication of clinical research and a completed CONSORT checklist must be included with all submissions.

Clinical trial registration	University of Helsinki: Dnro 91/13/03/00/2011), Heinrich Heine University Duesseldorf :3647/2011, King's College London: 11/H0802/6).
Study protocol	The study protocol is described in Materials and Methods.
Data collection	Patients and healthy volunteers were recruited to the clinical centers at the University of Helsinki, Helsinki, Finland, the Heinrich Heine university Duesseldorf, Duesseldorf, Germany, and at King's College London, London, UK between September 2011 and September 2012.
Outcomes	We anticipated significantly distinct skin microbiomes and transcriptomes in PSO and AD nonlesional and lesional skin compared to the normal skin in healthy volunteers. The microbiome was analysed by 16s rRNA gene sequencing and WGS, and the skin transcriptome by microarrays. We identified distinct microbial communities coupled to disease relevant patterns of cutaneous gene expression in the two patient groups, identifying potential biomarkers for future diagnostics and targets of therapy.

October 2018

This checklist template is licensed under a Creative Commons Attribution 4.0 International License, which permits use, sharing, adaptation, distribution and reproduction in any medium or format, as long as you give appropriate credit to the original author(s) and the source, provide a link to the Creative Commons license, and indicate if changes were made. The images or other third party material in this article are included in the article's Creative Commons license, unless indicated otherwise in a credit line to the material. If material is not included in the article's Creative Commons license and your intended use is not permitted by statutory regulation or exceeds the permitted use, you will need to obtain permission directly from the copyright holder. To view a copy of this license, visit http://creativecommons.org/licenses/by/4.0/
